# Genes of the Mitochondrial Apoptotic Pathway in *Mytilus galloprovincialis*

**DOI:** 10.1371/journal.pone.0061502

**Published:** 2013-04-23

**Authors:** Noelia Estévez-Calvar, Alejandro Romero, Antonio Figueras, Beatriz Novoa

**Affiliations:** Instituto de Investigaciones Marinas (IIM), Consejo Superior de Investigaciones Científicas (CSIC), Vigo, Spain; The Centre for Research and Technology, Hellas, Greece

## Abstract

Bivalves play vital roles in marine, brackish, freshwater and terrestrial habitats. In recent years, these ecosystems have become affected through anthropogenic activities. The ecological success of marine bivalves is based on the ability to modify their physiological functions in response to environmental changes. One of the most important mechanisms involved in adaptive responses to environmental and biological stresses is apoptosis, which has been scarcely studied in mollusks, although the final consequence of this process, DNA fragmentation, has been frequently used for pollution monitoring. Environmental stressors induce apoptosis in molluscan cells via an intrinsic pathway. Many of the proteins involved in vertebrate apoptosis have been recognized in model invertebrates; however, this process might not be universally conserved. *Mytilus galloprovincialis* is presented here as a new model to study the linkage between molecular mechanisms that mediate apoptosis and marine bivalve ecological adaptations. Therefore, it is strictly necessary to identify the key elements involved in bivalve apoptosis. In the present study, six mitochondrial apoptotic-related genes were characterized, and their gene expression profiles following UV irradiation were evaluated. This is the first step for the development of potential biomarkers to assess the biological responses of marine organisms to stress. The results confirmed that apoptosis and, more specifically, the expression of the genes involved in this process can be used to assess the biological responses of marine organisms to stress.

## Introduction

Apoptosis is an essential biological process in invertebrates for the development and maintenance of homeostasis [1]. Moreover, apoptosis is an important mechanism in adaptive responses to environmental stress [2] and could be responsible for the ecological success of organisms, reflecting the ability to modify different physiological functions when the environment imposes strict conditions. Apoptotic cell death has been primarily investigated in vertebrates and model invertebrates [3]–[9] but scarcely in mollusks [10]–[12]. There are two major apoptotic pathways: the extrinsic pathway and the intrinsic or mitochondrial pathway [3]. Many of the proteins involved in vertebrate apoptosis have also been identified in invertebrates, although several differences exist between these organisms [13].

In recent years, increasing genomic and proteomic data have revealed many new genes and protein sequences related to apoptosis [14], [15], particularly in the genus *Mytilus* [16]–[20]. Romero et al [21] used the transcriptomic information obtained from the MytiBase database [22] to identify initiator and executioner caspases in *M. galloprovincialis* and to analyze the modulation of these genes after exposure to UV light and different pollutants. However, most of the key genes involved in the intrinsic or mitochondrial apoptotic pathway have not been described in mollusks [10], [11]. In vertebrates, the Bcl-2 family proteins [23] and regulatory proteins, such as the Bax inhibitor-1 (BI-1) [24], tightly control the intrinsic apoptotic route. Bcl_2_ genes and the BI-1 gene have been characterized in non-model invertebrates [6], [25], [26], but there is no information regarding marine mollusks. In contrast, p53 is a well-described mitochondrial apoptotic gene in these animals and is considered as a marker of cellular stress in mussels [27]–[29].

In mammals, the hallmark of mitochondrial apoptosis is the permeabilization of the mitochondrial outer membrane (MOMP) and subsequent release of cytochrome c into the cytoplasm, which contributes to the formation of the apoptosome complex (Apaf-1/cyto-c/caspase 9), which induces caspase-3 [30]. Caspase-3 activates a heterodimeric protein, DNA fragmentation factor (DFF), which is responsible for the completion of DNA fragmentation, resulting in a laddering pattern [31].

To further characterize invertebrate apoptosis, particularly the intrinsic pathway of apoptosis in *M. galloprovincialis*, six newly described key genes associated with the mitochondrial apoptotic pathway were characterized in the present study. The modulation of the apoptotic process was characterized using selective inhibitors, and the expression of the newly characterized genes was evaluated through real-time PCR after UV light treatment with or without different apoptosis modulators.

## Materials and Methods

### Animals

Adult mussels *(Mytilus galloprovincialis)* were obtained from a shellfish farm located in Ria de Vigo (NW Spain). The animals were maintained in open circuit tanks with filtered and aerated seawater at 15°C and fed daily with *Isochrysis galbana*, *Tetraselmis suecica* and *Skeletonema costatum*. The bivalves were acclimatized for one week before the experiments. The CSIC National Committee on Bioethics reviewed and approved all experiments.

### Selection of Relevant Sequences and Amplification using RACE

Five sequences with high homology to genes involved in mitochondrial apoptosis and one sequence homolog to a DNA fragmentation-related protein were selected from the MytiBase database [22]. The following sequences and cluster Ids were selected: p53 tumor suppressor-like protein (p53) (MGC02732), p53 and DNA damage regulated protein (PDRP) (MGC08340), Bcl_2_ protein (MGC03747), Bcl-2-associated X protein (Bax) (MGC09152), Bax inhibitor-1 (BI-1) (MGC00186) and DNA fragmentation factor 45 (Dff-A) (MGC03626). The uncompleted sequences were finished using RACE according to the protocol of Romero et al [21]. The Primer3 (v. 0.4.0) software was used to design RACE primers (Table 1).

**Table 1:** Sequences of the primers used for RACE amplification and qPCR assays.

Name	Sequence
p53-RACE 5′-2	GGGGAACCATGGATGACAGTTCCAA
p53-RACE 5′-3	TTGGTGACCCATTCTGAGCCAGCTT
p53-RACE3′-1	CACCAGGAGGCAAAAAGAGGAAAGCA
p53_F1	GAATACTCTGGGAGAGGTCACAC
p53_R1	TTCTGAGCCAGCTTGAGGTATC
p53_F2	AATGTCACAAGCTTCAGTTTCAA
p53_R2	TAAGGTGGGGGTGATGTGAT
Bcl_2_-RACE5′-1	CCACAAATCCTTGCCAACCACCGTTA
qPCR p53-F	CTAGGTAGACGGGCAGTAGAAGTT
qPCR p53-R	GCCTCCTGGTGTTACTGTAGTGAT
qPCR PDRP-F1	CTGCCAAAGAAAGCTACAAAGAAG
qPCR PDRP-R1	CCTTTGACAATGGATTGAGGTT
qPCR Bax-F2	CCAACAGGTCCACCATTAGAAC
qPCR Bax-R2	CTCTTGGCCACAGTTAGGAATG
qPCR Bcl_2_-F1	AGATAACGGTGGTTGGCAAG
qPCR Bcl_2_-R1	TAACGCCATTGCGCCTAT
qPCR BI-1-F1	GGCCAGTTTTCTCACCTCCT
qPCR BI-1-R1	CCAATCCATGACTGGACCAA
qPCR Dff-A-F	GCTGCGTGTTGTTATAGCAGAG
qPCR Dff-A-R	CTTCACCTATGCCTTCAGGTCT
qPCR Actin-F	AACCGCCGCTTCTTCATCTTC
qPCR Actin-R	CCGTCTTGTCTGGTGGTA

### Structural and Phylogenetic Characterization

The nucleotide consensus sequences, translated proteins and subsequent homology comparisons were performed using previously described software tools [21]. The protein motifs and conserved domains in the complete open reading frames (ORF) were identified using SMART7 software and the PROSITE database. The transmembrane regions and ER membrane retention signals were predicted through the TMPred website and PSORT II software, respectively. The DNA binding sites, nuclear localization signals (NLS) and nuclear export signals (NES) were predicted through the PredictProtein, PredictNLS and the NetNES 1.1 servers, respectively.

The complete sequences were aligned with similar sequences from GenBank, and the phylogenetic trees were constructed by the NJ and ME methods [32] through MEGA software [33]. Nodal support was estimated using the same program with 10,000 bootstrap replicates. The starting sequences were also aligned using the MAFFT online server following and E-INS-i strategy [34]. The resulting alignment was pruned using Gblocks 0.91 b [35] and then was analysed using ProtTest 3.2 [36] to determine the best-fitting amino acid replacement model using the Akaike Information Criterion (AIC) [37], which was specified to estimate the maximum likelihood gene tree using PhyML 3.0 [38]. Nodal confidence was calculated using the aLRT method [39].

### Induction and Evaluation of Apoptosis in Mussel Hemocytes

The apoptotic process was induced in mussel hemocytes through UV-C light treatment (Sylvania G8T5 light bulb) for 45 min as previously described [21]. A control group, which was not exposed to UV light, was included in all experiments. Apoptosis was evaluated in irradiated hemocytes using confocal microscopy or flow cytometry.

The changes in the nuclear morphology and condensation of the chromatin were evaluated in hemocytes after exposure to UV light. Briefly, the hemolymph from individual mussels (500 µl of 10^6^ cells) was smeared onto glass slides. The cells were exposed to UV light, and at 3 and 24 h post-irradiation (pi), the hemocytes were stained with a DAPI solution (Sigma-Aldrich Co. LLC.) (0.1 ng/µl in filtered sea water-FSW), and the nuclear apoptotic changes were evaluated using a TSC SPE confocal microscope (Leica).

After irradiation, the apoptotic levels were measured in granulocytes and hyalinocytes (R1 and R2 regions, respectively), using flow cytometry as previously described [21]. The percentage of apoptotic and necrotic cells was quantified in 100,000 events using Annexin V-FITC and 7-Amino-Actinomycin (7-AAD).

### Induction of Reactive Oxygen Species (ROS) Production after UV Light Treatment

Three experiments were conducted to confirm the production of ROS after the UV light treatment in mussel hemocytes. Eight independent samples composed of pooled hemocytes extracted from three different animals were used in each experiment. The hemolymph was distributed in 24-well plates (BD), and the plates were incubated at 15 °C for 30 min for adhesion, washed with FSW and treated with UV light for 45 min. No irradiated absolute controls were maintained in the dark. The other cells were treated with 1 mL of N-acetyl-L-cystein (NAC) and pyrrolidine dithiocarbamate (PDTC) from Sigma (1 mM and 50 µM, respectively) for 1 h before UV treatment, as these components were scavengers of oxygen free radicals that directly interacted with reactive oxygen species. Immediately after 45 min irradiation, the production of ROS was measured using a 2′,7′-dichlorofluorescein-diacetate probe (DCFH-DA, Molecular Probes) through flow cytometry according to the protocol of Prado-Alvarez et al [40]. Cell Quest software (B&D) was used to determine the mean of fluorescence of 10,000 events in the R1 (granulocytes) and R2 (hyalinocytes) regions.

### Modulation of the Apoptotic Process Induced through UV Light Treatment

Five chemical reagents were selected to modulate, block or inhibit different stages of apoptotic cell death after UV irradiation, which included two chemical antioxidants, NAC and PDTC, to inhibit ROS production; pifithrin-α hydrobromide (PFT-α), a reversible inhibitor of p53-mediated apoptosis and p53-dependent gene transcription; the cell membrane stabilizer cyclosporin A (CsA), which blocks mitochondrial permeability transition pore formation and inhibits apoptosis; and the pan caspase inhibitor N-Benzyloxycarbonyl-Val-Ala-Asp (O-Me) fluoromethyl ketone (Z-VAD-FMK). All reagents were purchased from Sigma (Sigma-Aldrich Co. LLC.). The experiments were performed three times. Twelve samples consisting of hemocytes extracted from four different mussels were used in each trial. The cells were incubated for adhesion (30 min at 15°C), and the supernatants were replaced with 1 mL of FSW containing one of the inhibitors, NAC (1 mM), PDTC (50 µM), PFT-α (100 µM), CsA (20 µM) or Z-VAD-FMK (100 µM). The most suitable and non-toxic concentration of each reagent was selected after a toxicity evaluation of four serial dilutions of each compound. Toxicity evaluation was conducted on hemocytes treated for 1 h with different concentrations of the inhibitors; NAC (0.1, 1, 10 and 100 mM), PDTC (5, 50 and 500 µM), PFT (0.1, 1 and 100 mM), CsA (0.2, 2 and 20 µM), Z-VAD-FMK (1, 10 and 100 µM). The cell viability was measured 3 and 24 h after the treatment by flow cytometry using 7-AAD staining protocols. The control samples were only treated with FSW. The samples were incubated with inhibitors at 15 °C in the dark for 1 h. Subsequently, the cells were washed with FSW and irradiated for 45 min. The control plates were maintained at room temperature in the dark. After UV light treatment, the supernatants were discarded and 1 mL of fresh FSW was added.

### Gene Expression Analysis using Quantitative PCR

The analysis of the gene modulation was conducted in two independent experiments using three independent biological samples in each one consisting of pooled hemocytes extracted from four animals. The pooled hemocytes were distributed in 24-well plates (BD) and treated with UV light for 45 min. The cells treated with PFT-α (100 µM) or with FSW (controls) were sampled immediately after irradiation (0 h) and at 3, 6, 24 and 48 h post-irradiation. At each sampling, the supernatants were discarded and the hemocytes were scraped from the bottom of the well with 500 µl of TRIZOL Reagent (Invitrogen, USA). Total RNA extraction and cDNA synthesis were performed using the previously described standard protocols of Romero et al [21]. Specific qPCR primers were designed using Primer3 (v. 0.4.0) software according to known qPCR restrictions such as amplicon size, Tm difference between primers, G:C content, self-dimer or cross-dimer formation (Table 1). The efficiency of the primer pairs was analyzed in serial five-fold dilutions of cDNA by calculating the slope of the regression line of the cycle thresholds (Cts) versus the relative concentration of cDNA [41]. The following slope values were obtained: −2.91 for p53, −3.11 for PDRP, −3.19 for Bax, −3.05 for Bcl_2_, −3.12 for BI-1 and −3.17 for Dff-A. The PCR reactions were performed as technical triplicates. The cycle threshold (Ct) data were compared using the comparative Ct method (Ct) [41] and the geometric mean derived from the expression of mussel β-actin as housekeeping gene, which was constitutively expressed in mussel tissues and not affected by UV light treatment [21]. The fold-change units were calculated by dividing the normalized expression values in the UV-treated hemocytes by the normalized expression in the non-irradiated control cells.

### Statistical Analysis

All results are expressed as the mean ± SD of the different biological samples specified, and the data were analyzed using Student’s t-test. The differences were considered statistically significant at p<0.05.

## Results

### Structural and Phylogenetic Characterization

We selected five different genes from *M. galloprovincialis* that have been implicated in mitochondrial degradation and the consequent activation of the apoptotic process based on vertebrate and invertebrate models: p53, PDRP, Bcl_2_, Bax and BI-1. These genes were cloned and sequenced. In addition, a sequence with high identity to DNA fragmentation factor A (Dff-A) was selected. The length of the obtained sequences, the ORFs, the deduced amino acid sequences and the GenBank accession numbers are summarized in Table 2. The structure and predicted domains were characterized.

**Table 2:** GenBank accession numbers and the lengths of the selected sequences described in the text. (bp) length of sequences in base pairs.

Name	Sequence (bp)	ORF (bp)	Protein(aa)	GeneBank accession number
**p53**	2185	1308	435	KC545827
**PDRP**	746	390	129	KC545828
**Bcl_2_**	1172	558	185	KC545829
**Bax**	1175	630	209	KC545830
**BI-1**	863	711	236	KC545831
**Dff-A**	828	603	200	KC545832

(aa) length of proteins in number of amino acids.

#### p53

**Figure 1:**
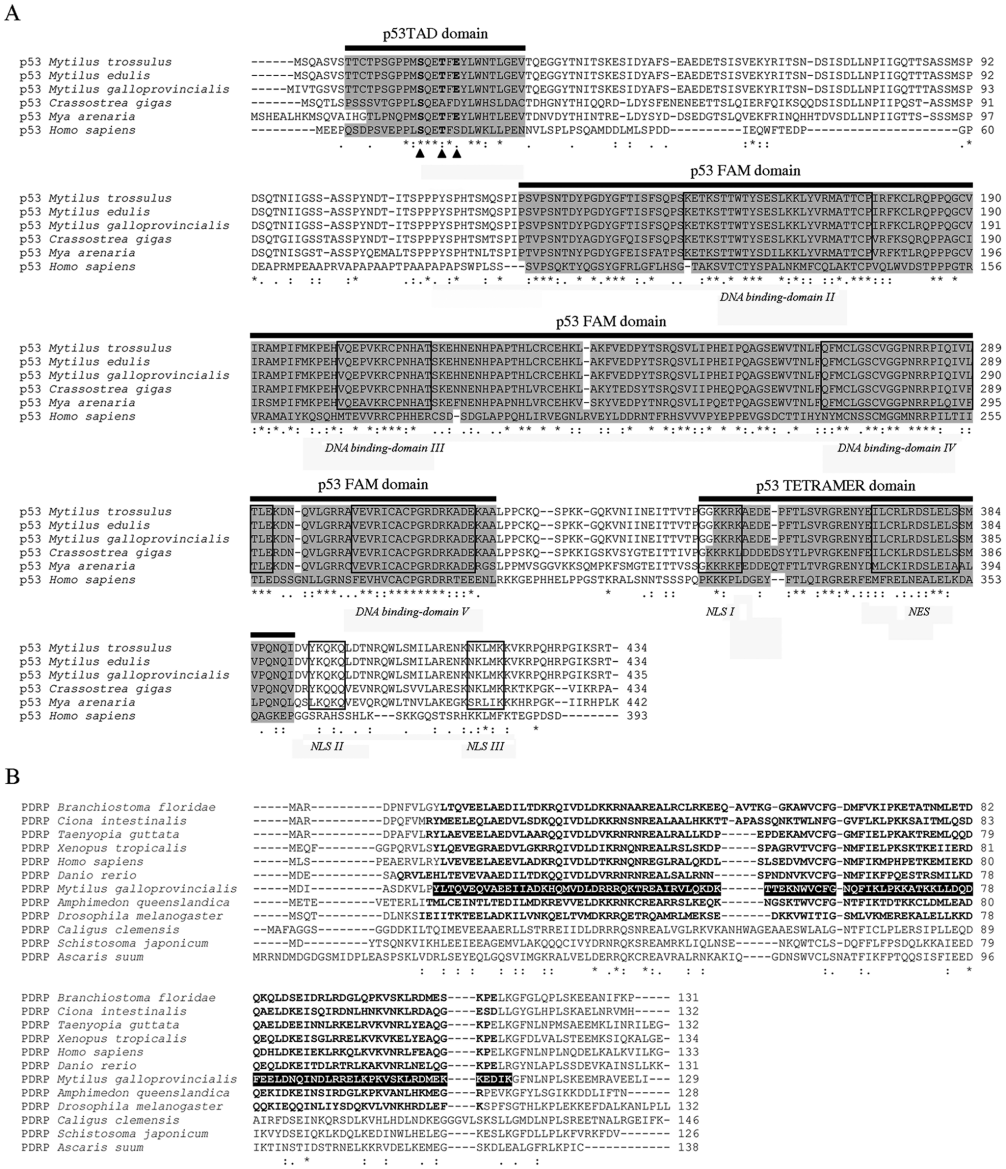
A: Structural characterization of the p53 protein. The three domains of p53 are highlighted. The black triangles show the conserved phosphorylation sites. The four DNA-binding domains are boxed. The TETRAMER domain contains the NES. NLS I, NLS II and NLS III refer to the three nuclear localization signals. **B: Structural characterization of the PDRP protein.** Only a Prefoldin 2 domain is present. These domains are indicated in bold letters, and the location of this domain in *M. galloprovincialis* is highlighted.

The p53 gene from *M. galloprovincialis* displayed the highest identity with similar sequences from *M. edulis* and *M. trossulus* (up to 98%). The deduced protein showed a multidomain structure containing TAD, FAM and TETRAMER domains (Figure 1A). All essential amino acids from the Mdm2 binding site (F, L, W, L) and phosphorylation sites (S, T and E) were observed within the TAD domain. The p53 protein contained five DNA binding domains. A nuclear export signal (NES) was identified within the TETRAMER domain, and three nuclear localization signals (NLS I, II and III) were located between residues 349–354, 394–398 and 414–416, respectively (Figure 1A). A phylogenetic analysis of p53 was performed with a total of 17 sequences from vertebrates and invertebrates. The p53 sequences from different mollusks were consistently grouped into one clade, which was more related to chordates than to other invertebrates (81% of bootstrap value) (Figure S1A).

#### PDRP

The PDRP gene from mussels showed the highest identity to a similar sequence from *Taeniopygia guttata* (E-value of 3e^−35^). Structurally, only a Prefoldin 2 domain was predicted between amino acids 11 and 110 (Figure 1B). The phylogenetic tree of PDRP grouped the sequence from *M. galloprovincialis* in the central part of the tree, closer to chordates than to other invertebrates, such as arthropods, platyhelminthes and nematodes, although without high confidence (Figure S1B).

#### The Bcl-2 family members

**Figure 2:**
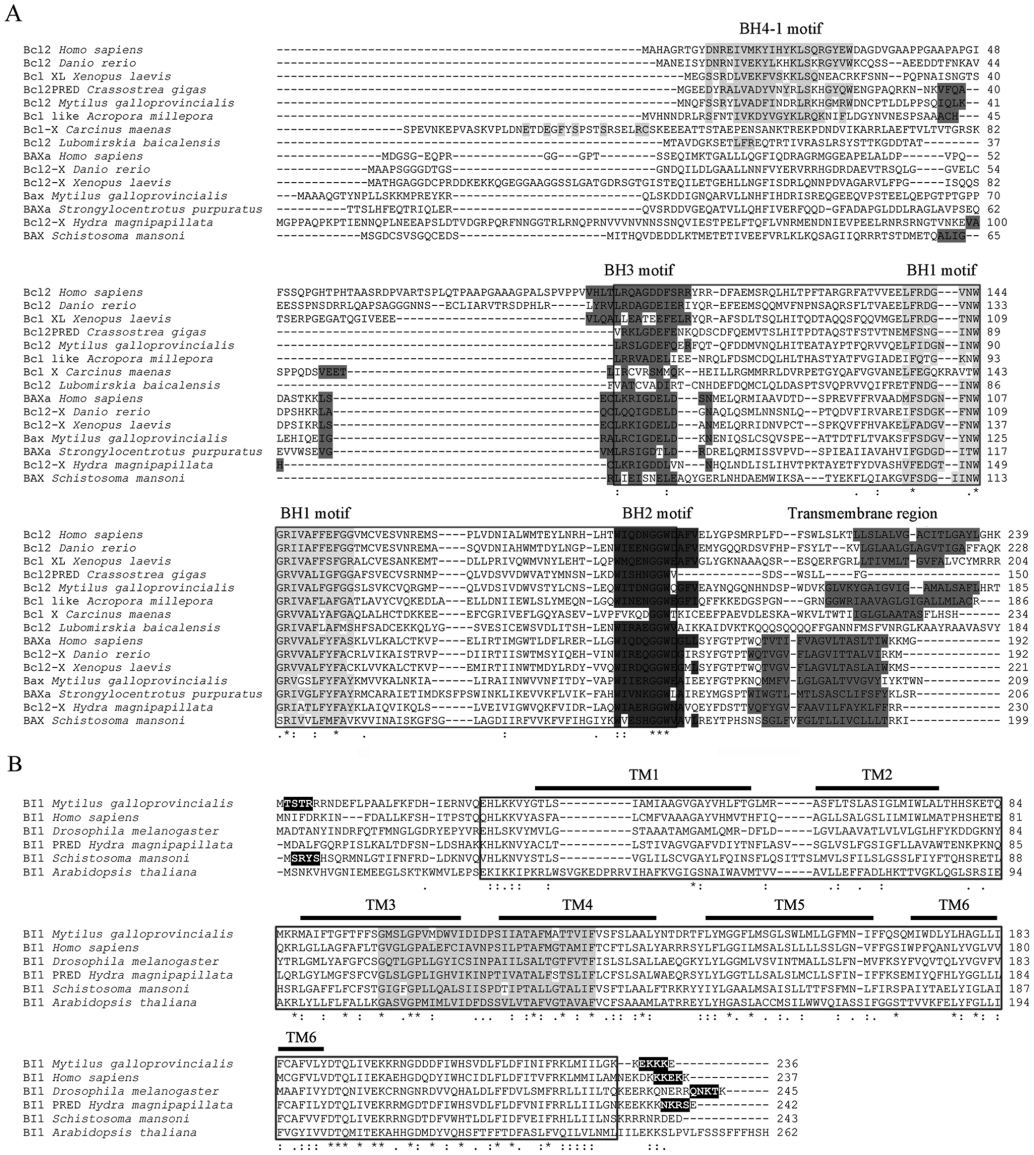
A: Structural characterization of the Bcl-2 family proteins. The Bcl domain is boxed. The four BH regions and the transmembrane domain are indicated in gray. **B:** **Structural characterization of the BI-1 protein.** The BI-1 domain is boxed, and the family signature is highlighted. The six transmembrane domains are indicated. Double ER membrane retention signals are highlighted in black.

Bcl_2_ and Bax sequences described in *M. galloprovincialis* showed the highest identities with a predicted Bcl_2_-like protein from *Saccoglossus kowalevskii* (E-value of 2e^−51^) and a Bax protein from *Ictalurus punctatus* (E-value of 8e^−36^), respectively. A Bcl domain containing four and three BH regions was predicted in Bcl_2_ and Bax proteins, respectively. In addition, transmembrane domains were predicted in the C-terminal region of both proteins (Figure 2A). The phylogenetic tree constructed using 20 proteins from the Bcl-2 family separated the Bcl_2_ and Bax sequences into two different clades: Group I and Group II, respectively. The Bcl_2_ from mussel was located within Group I in the same branch with *Crassostrea gigas* (Figure S2A). The mollusk Bcl_2_ was located closer to vertebrates than to other invertebrates, such as arthropods, cnidarians or sponges. A similar relationship was observed in Group II, where the Bax protein from mussels was placed closer to vertebrates than to other invertebrates (Figure S2A).

#### The BI-1 protein

The BI-1 gene from mussels showed the highest identity with that of *Salmo salar* (E-value 1e^−82^). A unique BI-1 domain was located between residues 28 and 230 and contained six predicted transmembrane domains (Figure 2B). The conserved BI-1 signature motif (G-x(2)-[LIVM]-[GC]-P-x-[LI]-x(4)-[SAGDT]-x(4,6)-[LIVM](2)-x(2)-A-x(2)-[MG]-T-x-[LIVM]-x-F) was located between residues 99 and 128. Two ER retention signals were predicted in the N- and C-terminus of the sequence (XXRR-like and EKKK motifs, respectively) (Figure 2B).

A phylogenetic analysis was constructed including representatives of the six different groups of proteins containing the transmembrane BI-1 motif (TMBIM) (Figure S2B). Two solid clusters were observed. The first cluster included the BI-1 proteins and TMBIM5 members, and the second cluster included the four TMBIM proteins (1 to 4). The BI-1 from mussels was located closer to similar proteins from vertebrates, instead of other basal taxonomic groups, such as arthropods or platyhelminthes (Figure S2B).

#### The Dff-A protein

**Figure 3:**
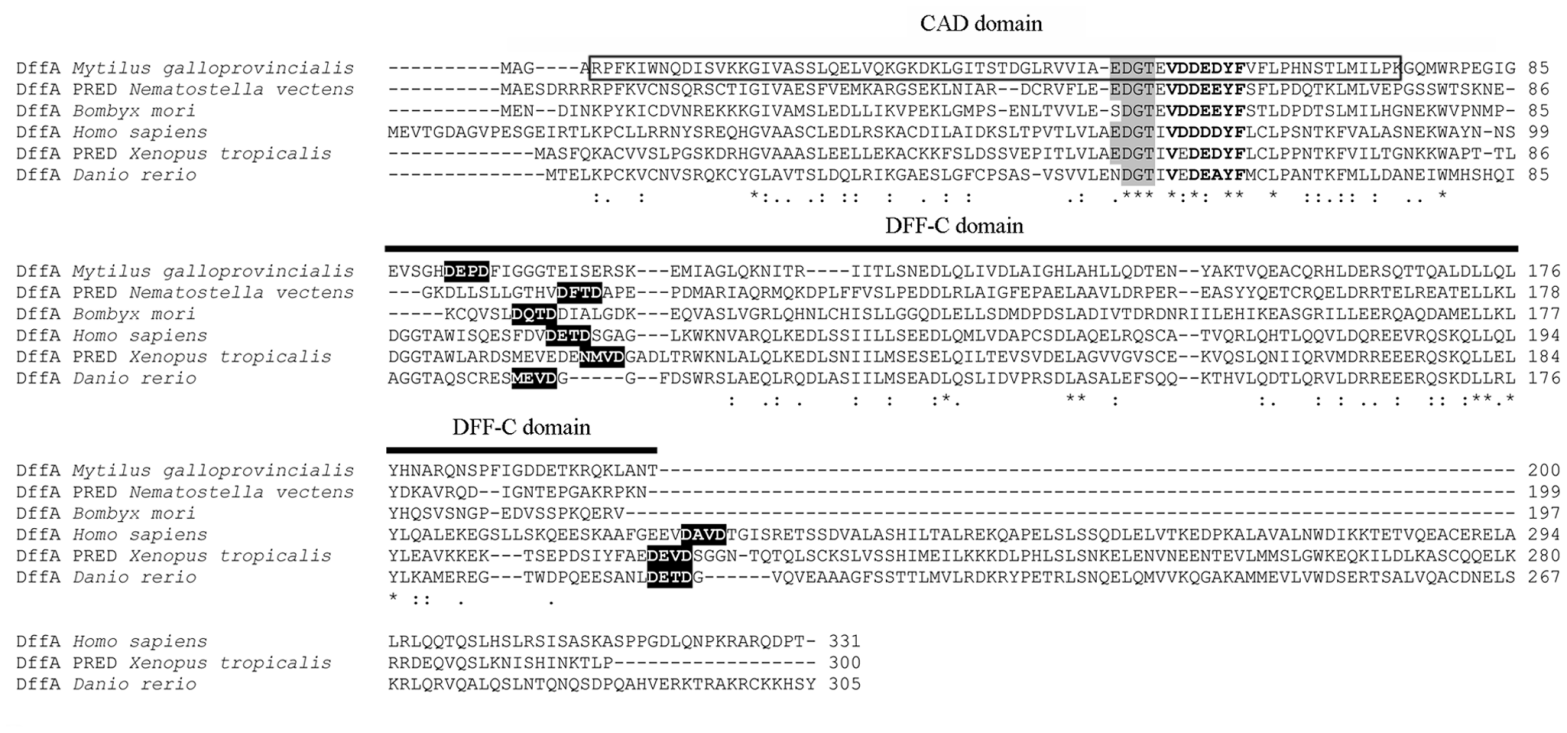
Structural characterization of the Dff-A protein. Alignment of Dff-A proteins from *M. galloprovincialis*, *N. vectensis*, *B. mori*, *H. sapiens*, *X. tropicalis* and *D. rerio.* The CAD and DFF-C domains are indicated. The CAD domain is boxed, and the EDGT motif is highlighted in gray. The conserved signature VDDXXYF is indicated in bold letters. The predicted positions for the caspase cleavage sites are highlighted in black.

The Dff-A sequence from mussels exhibited the highest similarity with that of *Saccoglossus kowalevskii* (E-value of 1e^−36^). The Dff-A protein contained CAD and C-terminal DFF-C domains. The CAD domain was located between residues 5 and 79 and included two conserved signature motifs (EDGT and VDDXXYF). The DFF-C domain between residues 85 and 200 included the caspase cleavage site (Figure 3). A phylogenetic tree was used to represent the relationship between the Dff-A sequences from *M. galloprovincialis* and several representative taxonomic groups of the CIDE, Dff-A and Dff-B families (Figure S3). This phylogenetic tree divided all of the sequences into main branches: the CIDE, Dff-A and Dff-B families. The Dff-A sequence from mussels was grouped with similar proteins from other invertebrates, such as arthropods and cnidarians, and far from chordates (bootstrap value of 80%) (Figure S3).

**Figure 4:**
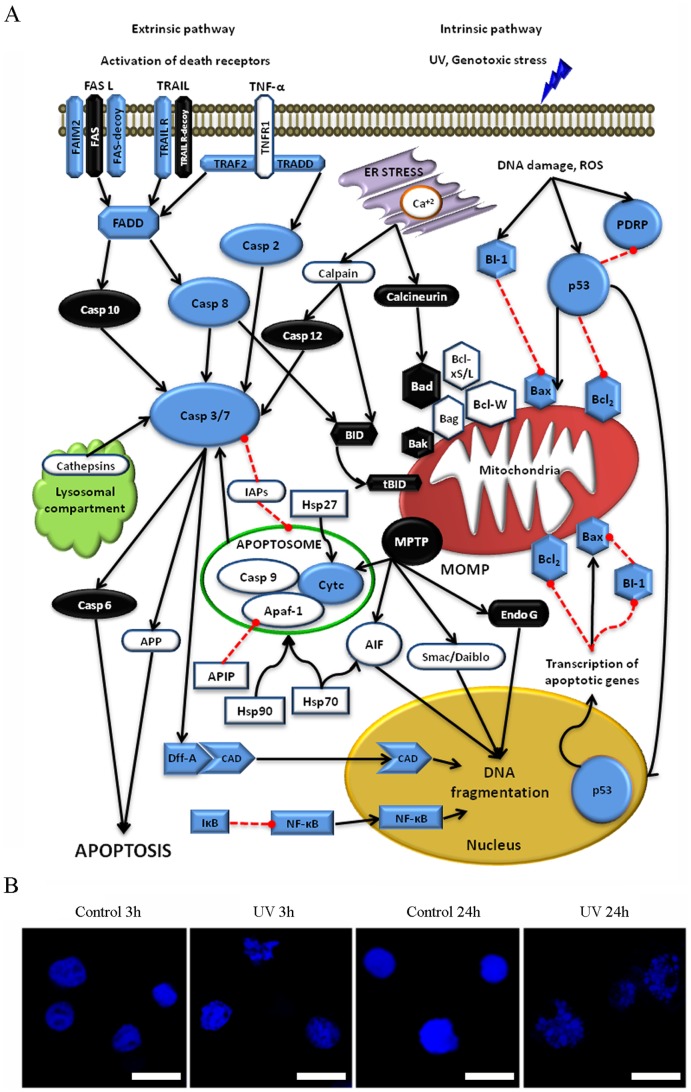
A: Schema representing the major apoptotic pathways in mollusks. The blue symbols indicate the genes described in bivalves [20], [21], [30], [31], [37], [65]–[69], [84], [89]–[102]. The white symbols indicate the available ESTs, and the black symbols indicate genes that have not been described in mollusks. The red lines indicate inhibition. **B**: M**orphological changes in mussel hemocytes after UV-induced apoptosis**. The nuclear morphology and chromatin condensation was evaluated using confocal microscopy at 3 and 24 h after irradiation. Scale bar 7.5 µm.

The characterization of these genes facilitated the construction of a general overview of the mitochondrial apoptotic pathway in mollusks. The compiled information in the molluscan apoptotic map was based on the genes characterized in the present study, apoptotic-related ESTs from genomic databases and an extensive revision of the bibliography (Figure 4A). We confirmed that the two major apoptotic pathways described in vertebrates were also present in bivalves. The extrinsic pathway is initiated through the activation of death receptors. Activated receptors recruit FADD molecules and cleave caspase 8, which activates effector caspases. Four different executioner caspases have been previously described in mussels, and several IAPs have been identified in clams and oysters. The intrinsic pathway is initiated through stimuli that induce DNA damage, such as UV light. The regulatory activity of PDRP and BI-1 proteins, the participation of the p53 and Bcl-2 proteins in the MOMP and the release of the cyto-c to the cytosol are characteristics of this pathway. Although scarce information is available regarding caspase-independent apoptosis in bivalves and the involvement of the ER and lysosomes in apoptosis has not been analyzed, several cathepsins and calpains have been identified in clams and mussels. Regardless of the pathway, executioner caspases induce the fragmentation of DNA and the formation of apoptotic bodies through the activation of DFF proteins and their subsequent translocation to the nucleus. The result of this process can be observed after mussel hemocytes are treated with UV light (Figure 4B). As early as 3 h after UV treatment, the characteristic features of chromatin condensation were detected in the nucleus of some irradiated cells. After 24 h, almost all cells presented apoptotic changes. The nucleus presented an irregular morphology and consisted of a high number of fragments of condensed chromatin (Figure 4B).

### Modulation of the Apoptosis Intrinsic Pathway in Mussel Hemocytes

**Figure 5:**
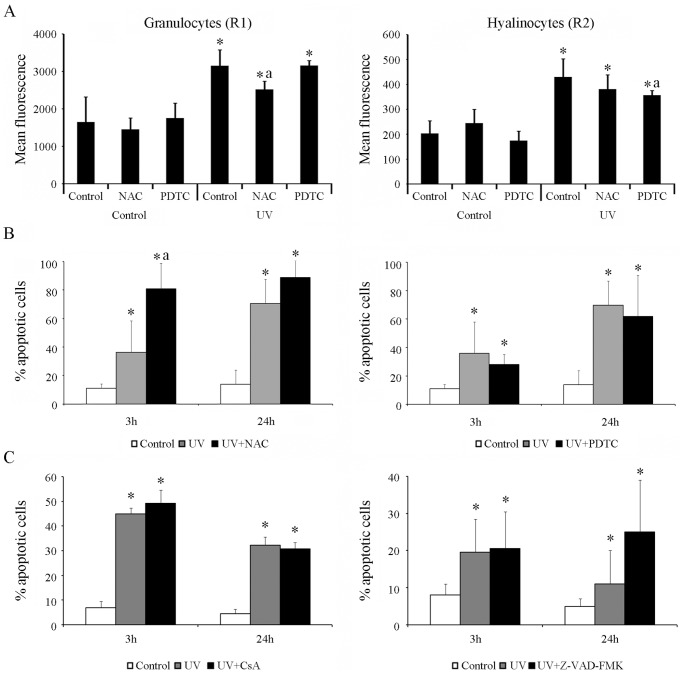
Modulation of apoptosis induced through UV light in mussel hemocytes. **A:** Quantification of the ROS production through the measurement of the mean fluorescence in granulocytes (R1 region) and hyalinocytes (R2 region) immediately after irradiation. The results are shown as the mean ± SD of eight biological samples **B**: Effects of antioxidant treatments (NAC and PDTC) before the induction of apoptosis. **C**: Analysis of apoptosis levels after treatments with CsA and ZVAD-FMK. The results in B and C are shown as the mean ± SD of twelve biological samples. (*) Significant differences compared with non-irradiated control cells. (a) Significant differences compared with UV-treated control cells.

The UV light treatment in mussel hemocytes induced the production of ROS in granulocytes and hyalinocytes (R1 and R2 regions, respectively) (Figure 5A). Although a significant increase of the mean fluorescence levels was registered in both cellular types, the granulocytes showed 10-fold higher levels than the values observed in hyalinocytes (mean fluorescence levels ranged from 357 to 429 in hyalinocytes and 2519 to 3154 in granulocytes). Moreover, treatment with the antioxidants NAC or PDTC induced a significant reduction of ROS production in granulocytes and hyalinocytes, respectively after UV treatment (significant reductions marked with “a” in Figure 5A). However, treatment with these two antioxidants did not induce a significant reduction of the apoptotic levels at any time, regardless of the cell population analyzed. In contrast, the treatment with NAC induced a significant increase in the percentage of apoptotic cells at 3 h after irradiation, reaching values of up to 80% (Figure 5B). Neither the treatment with the cell membrane stabilizer CsA nor with the caspase inhibitor Z-VAD-FMK significantly reduced the percentage of apoptotic cells compared with the values registered in untreated hemocytes at 3 and 24 h after the UV treatment (Figure 5C).

**Figure 6:**
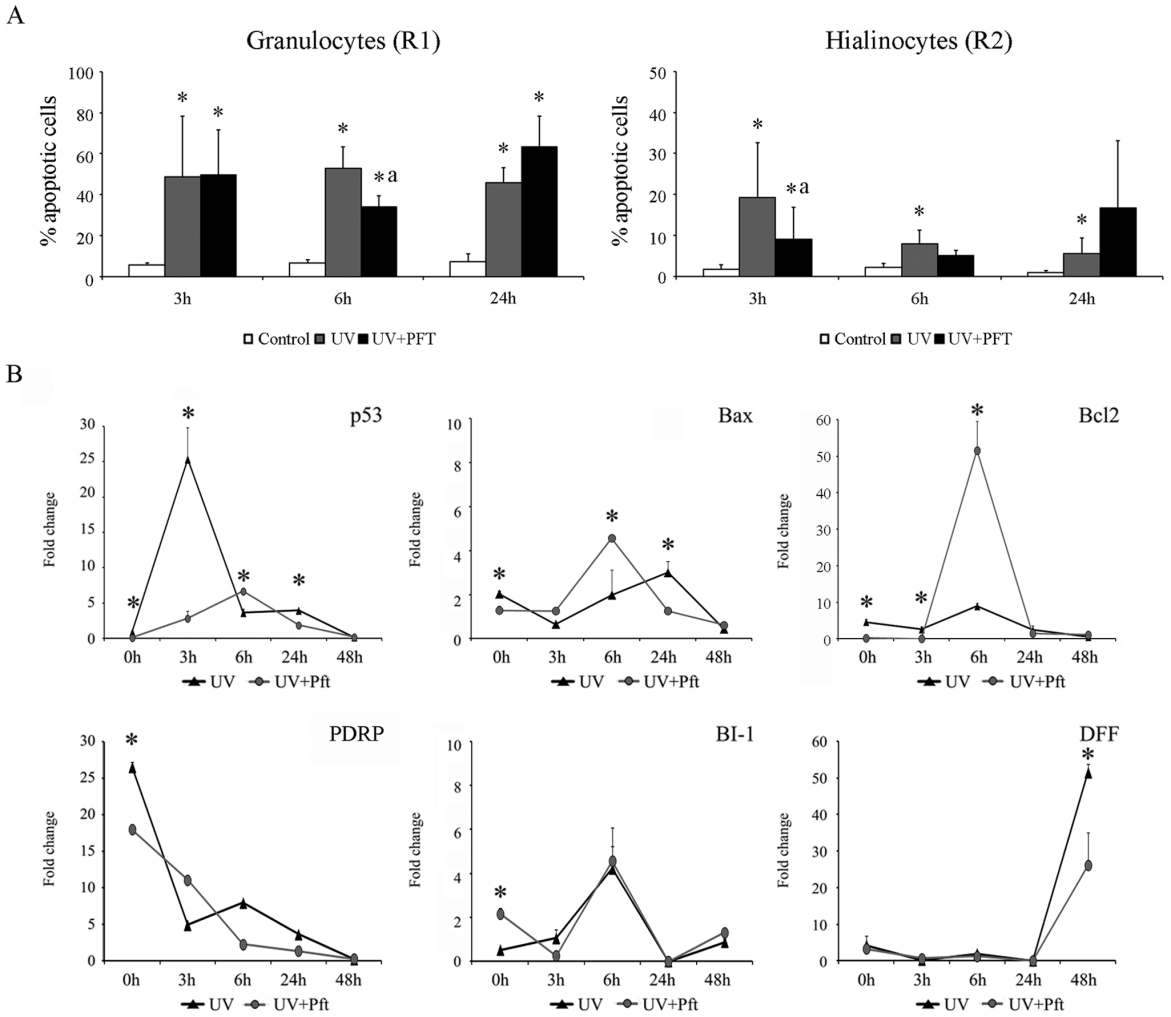
A: Apoptotic levels in hemocytes treated with PFT-α prior to UV irradiation. Apoptotic levels measured at 3, 6 and 24 h post-irradiation. The results are shown as the mean ± SD of twelve biological samples. (*) Significant differences compared with non-irradiated control cells. (a) Significant differences compared with irradiated control cells. **B: Gene expression profile in hemocytes treated with PFT-α.** The expression of p53, Bax, Bcl_2_, PDRP, BI-1 and Dff-A genes was quantified after 0, 3, 6, 24 and 48 h pi. The results are shown as the mean ± SD of the three pools used in these experiments. (*) Significant differences compared with the non-treated cells at each sampling point (p<0.05).

Only treatment with the reversible inhibitor of p53-mediated apoptosis, PFT-α, induced a significant reduction of the apoptotic levels. The reduction was observed in hyalinocytes and granulocytes at 3 and 6 h after UV treatment, respectively, compared with the values registered in irradiated control cells. A decrease from 19% to 9% was observed in hyalinocytes and from 52% to 34% in granulocytes. No reduction in the apoptotic levels was registered at 24 h (Figure 6A).

To understand the molecular mechanisms underlying the reduction of the apoptotic levels after the treatment with PFT-α, the expression of selected key genes was analyzed using qPCR. The PFT-α treatment modulated, reduced and delayed the expression of the different genes (Figure 6B). The expression of the p53 gene was highly modulated through UV treatment. The irradiated cells showed a maximum expression level of up to 25 times more than that in the control cells at 3 h pt. The expression was reduced to basal levels by the end of the experiment, and treatment with PFT-α modified these kinetics. At 3 h pt, the expression in PFT-treated cells was only 2 times more than that in the control cells. Moreover, the maximum expression level was delayed until 6 h pt (only 6 times more than the control level) (Figure 6B). The PDRP gene was highly up-regulated immediately after irradiation (0 h), reaching values of up to 27 times more than in the control. The PFT-α treatment significantly reduced this expression to 18 times more than in the control. The expression levels were reduced until 48 h, when no significant differences were observed (Figure 6B). Treatment with PFT-α significantly up-regulated the expression of the anti-apoptotic genes BI-1 and Bcl_2_ at 0 and 6 h pt, respectively. Meanwhile, the fold-change observed in the BI-1 gene was low (only 2 times more than in the control at 0 h pt), and the Bcl_2_ gene expression reached significant values of up to 36 times more than in the control at 6 h pt. Treatment with PFT-α did not affect the kinetics of BI-1 expression, and the expression levels increased until reaching maximum levels at 6 h pt (4 times more than the control level) and gradually diminished to the basal levels at 48 h (Figure 6B). The pro-apoptotic gene Bax was also modulated through PFT treatment. The PFT-treated cells reached the highest significant values (4.5 times more than in the control) at 6 h pt, while the non-treated cells reached significant values at 24 h pt (3 times more than in the controls). At 48 h pt, no significant differences were observed (Figure 6B). Moreover, the expression of the Dff-A gene, as a marker for the later stages of apoptosis, was significantly up-regulated at 48 h after irradiation, and the PFT treatment induced a significant reduction of expression from a 52- to a 26-fold increase in PFT-α-treated cells (Figure 6B).

## Discussion

In the present study, six newly described apoptotic genes were characterized in *M. galloprovincialis* (p53, PDRP, Bcl2, Bax, BI-1 and Dff-A) to determine the molecular mechanisms of the mitochondrial pathway in bivalves.

The p53 gene is a connection point of several stress response pathways and governs the decision between cell survival and apoptosis [42]. In mussels, this gene showed structural features similar to those previously described in humans and other bivalves [29], [43]. The presence of DNA binding domains, the proline-rich region, the Mdm2 binding site, NLS and NES suggests the involvement of transcriptional and non-transcriptional mechanisms of activation and regulation, similar to vertebrates [44], [45]. The PDRP gene is a downstream target gene of p53 [46], which had not been formally described in invertebrates. Our analysis revealed that the mussel protein contains a conserved Prefoldin 2 motif that is present in chordates and some invertebrates, such as sponges and cnidarians. Although the function of PDRP in apoptosis is not currently known, this protein has been implicated in protein-protein and protein-DNA interactions [46]. The mussel Bcl_2_ and Bax genes, considered as key regulators of this process, contain the characteristic BH domains and transmembrane regions [23], [47]. The newly described mussel BI-1 contained six transmembrane domains and a typical signature motif similar to humans [48]. Moreover, two ER retention signals were identified, suggesting a potential ER localization, where BI-1 could exert its anti-apoptotic activity, as described in vertebrates [24], [48]. The Dff-A gene functions at terminal stages of apoptosis, inducing chromatin condensation and DNA breakdown [31]. The deduced Dff-A protein from mussels contained the signature motif for this family, the caspase cleavage site and nuclease domain at the DFF domain, which distinguishes Dff-A from CIDE family proteins [49].

The phylogenetic analysis revealed that the components of the mitochondrial apoptotic pathway in *M. galloprovincialis* are more associated with vertebrates than with other invertebrates. Similar results have been observed using several approaches. The unexpected phylogenetic position of bivalve apoptotic genes closer to vertebrate sequences has been previously described for p53 [27], [29], [50], with a separation between the lineages of mollusks, chordates and other animals, such as tunicates, echinoderms and arthropods. Moreover, different components of the Bcl-2 family proteins in cnidarians have also been reported as closer to vertebrate sequences than to invertebrates [51]. This divergence and phylogenetic position of the mussel sequences were also observed in our analysis, not only for p53 or BI-1 proteins, which are well represented in all taxonomic groups, but also for the PDRP gene and Bcl-2 family members for which there were not enough invertebrate sequences to obtain a highly confident phylogenetic analysis. This situation potentially reflects the different degrees of divergence in the mitochondrial apoptotic genes in different animal phyla [52], as the mitochondrial apoptotic pathway arose before the emergence of the deuterostomes, and all or portions of the pathway have been lost in some lineages [53], [54]. In contrast, the mussel Dff-A gene, which is not exclusively associated with the intrinsic pathway, was closer to homolog sequences from cnidarians and insects than to vertebrates because this gene has not been described in urochordates, echinoderms and nematodes [55].

The representation of the apoptotic framework, including ESTs and genes described in bivalves, indicates that apoptosis in this group has a complexity similar to vertebrates. Apoptosis in mollusks acts through extrinsic or intrinsic pathways [12]. A variety of death receptors, the Fas-associated death domain (FADD) and caspase 8 have been described in mollusks [21], [56], [57]. The association between the extrinsic and intrinsic pathways in vertebrates is mediated through the Bid gene, a BH3-only protein, which indirectly activates executioner caspases [58]. Interestingly, to date, no homolog sequences of the BH3-only proteins have been detected in bivalves using high throughput sequencing technologies [56], [57], and these genes are not represented in the MytiBase database [22], suggesting that alternative associations between intrinsic and extrinsic pathways could exist in bivalves. The central hallmark of the mitochondrial apoptotic pathway in vertebrates is the induction of MOMP, the release of cyto-c and the formation of an apoptosome complex (Apaf-1/cyto-c/caspase 9) that directly induces caspase 3 [30]. Notably, apaf-1 and caspase 9 genes have not been formally described in bivalves, and only automatically annotated sequences for caspase 9 have been identified in oysters and Manila clams [56], [59]. Other factors released from the mitochondria, such as SMAC/Diablo or AIF-like genes, have been identified in oysters [59]. Bcl-2 family proteins control MOMP in vertebrates [23]. Various members of this family, such as Bag, Bcl-xL and Bcl-xS, have recently been described in oysters and mussels [59], [60].

In this study, UV light was used because it induces a complex apoptotic mechanism in which a variety of pathways are involved [61]. First, we determined whether UV irradiation enhances ROS production in mussel hemocytes in a manner similar to that described in mammals [62]. In mussels, the UV treatment induced ROS production in hemocytes. Flow cytometry revealed that granulocytes were the most active, while the hyalinocytes also showed a certain level of oxidative activity, but to a lesser extent. Although the differences in ROS production between the cell types have been documented in oysters [63], [64] or clams [40], to our knowledge, this is the first report of ROS production in mussel hemocytes after UV stimulation.

ROS are involved in the oxidation of mitochondrial pores for the release of cyto-c [65]. To determine whether ROS are involved in UV-induced apoptosis, the effects of two radical scavengers were assessed. The treatment of hemocytes with these antioxidants did not affect apoptosis levels, although both NAC and PDTC have been implicated in the reduction of apoptosis in UV-treated HeLa cells [62]. These results suggest that ROS could not be directly involved in the UV-induced apoptotic process in bivalves, although the induction of severe cell damage is impossible to overcome with any antioxidant.

The analysis of apoptotic gene expression using qPCR revealed that the genes characterized here were modulated after UV radiation. The p53 gene, considered as a major mediator of the UV-induced stress response [61], was highly expressed in hemocytes at 3 h pi. This high p53 expression level was expected, as UV radiation induces the expression of the p53 gene in a dose-dependent manner [66]. Although a novel non-transcriptional role for p53 has been identified in molluscan apoptosis [28], [29], this gene could act as a transcription factor after translocation to the nucleus to regulate the expression of downstream target genes [44], such as PDRP [46]. In mussel hemocytes, the PDRP gene is an early-responsive gene. At 0 h, when p53 was not expressed, the UV treatment induced the up-regulation of the PDRP gene in a p53-independent manner. However, at 3 h, the PDRP gene was down-regulated while the p53 gene reached its maximum level. The modulation of the PDRP gene, probably induced by the p53 gene, was consistent with previous results obtained in humans [46]. In our animal model, the p53 gene also could induce the up-regulation of the pro-apoptotic Bax gene at 24 h pi and simultaneously it could repress the transcription of the anti-apoptotic genes Bcl_2_ and BI-1. This regulation is consistent with previous observations in mammals [44], [67]. As expected, the Dff-A gene was highly up-regulated at the last sampling point, as Dff-A induces DNA breakdown [31]. Based on the results obtained in this study, it is also possible that a transcription-independent p53-mediated pathway exists in bivalves, although the exact mechanisms underlying the modulation of the PDRP, Bax, Bcl_2_ and BI-1 genes in mussels are unknown. In mammals, this pathway is induced through the accumulation of p53 in the cytosol or mitochondria and the direct activation of Bax protein [68]. It is important to remind that the activity of members of the Bcl-2 protein family (including Bcl_2_ and Bax) depends on post-translational modification (e.g. proteolysis, phosphorylation, conformational changes, etc.), in combination with sub-cellular re-localization and protein-protein interactions.

To address the relative contribution of UV irradiation and p53 to mitochondrial apoptosis, the apoptosis level and gene expression were analyzed in the presence of PFT-α, a chemical compound that selectively blocks the potential transactivation of p53 and p53 downstream genes in a variety of cell types [69], [70].

The PFT-α treatment in mussel hemocytes reduced apoptotic levels at short time periods after UV exposure; however, no inhibitory effect was observed at 24 h. A similar response was reported in CS-B human cells, where PFT-α did not exert any effect at times long after UV treatment [69], suggesting that transcriptional mechanisms are more significant at short times and non-transcriptional mechanisms are relevant at long times, although the effect of PFT-α on the induction of apoptosis in a particular cell type is highly dependent on the relative importance of pro- and anti-apoptotic genes induced through p53 [71]. The treatment of hemocytes with PFT-α induced not only the down-regulation of p53 and PDRP at 3 h pi but also the up-regulation of Bax and Bcl_2_ genes at 6 h, suggesting the participation of these genes in non-transcriptional mechanisms that occur at 6 h. The significant increase in Bcl_2_ expression could occur in response to the pro-apoptotic effect of Bax [72]. Interestingly, the down-regulation of Dff-A gene in presence of PFT-α at 48 h suggested that its expression could be dependent on the potential transactivation of the p53 gene.

To analyze the implication of the MOMP in the apoptosis induced by UV light in mussel hemocytes, the cells were treated with CsA, which blocks mitochondrial permeability transition pores and thereby inhibits apoptosis in a wide variety of cell types [73]–[74]. In mussel hemocytes, CsA treatment did not exert any substantial effect on apoptosis. The most feasible hypothesis, as previously proposed for other invertebrate models, could be that membrane depolarization is not required for the liberation of pro-apoptotic factors into the cytosol [13], [53], [75].

Mitochondria control the apoptotic process through caspase activation and caspase-independent mechanisms [76]. The general caspase-inhibitor Z-VAD-FMK was used to analyze the activity of mussel caspases in the intrinsic apoptotic pathway. The treatment of mussel hemocytes with this inhibitor did not prevent cell death, although Z-VAD-FMK inhibited noradrenalin-induced apoptosis in *Crassostrea gigas* hemocytes [77]. This result suggests that different genotoxic stresses can trigger diverse apoptotic molecular mechanisms, even in similar organisms. The lack of significant effects of this inhibitor on apoptosis has been extensively described, suggesting that a caspase-independent pathway could direct the observed cell death [78], [79].

In recent years, the levels of pollutants in marine ecosystems have increased to alarming rates [80]. The biological responses of fish and marine invertebrates such as mussels have been used to analyze the impact of contaminants [81]–[84]. New biological tools or biomarkers are currently being developed [85] to evaluate the impact of pollutants in indicator species through the measurement of sub-lethal effects [86], [87]. The evaluation of the DNA integrity after exposure to pollutants is an indirect measurement of the apoptotic process [88].

Despite the complexity of the intrinsic apoptosis pathway, this study has provided new information concerning the molecular mechanisms that lead to apoptotic cell death in bivalves. The results of this study are of great interest not only for the development of potential biomarkers to assess the biological responses of marine organisms to stress but also for the study of other aspects such as cellular physiology, development, or pathogen/host interactions.

## Supporting Information

Figure S1 Phylogenetic relationship of p53 (A) and PDRP1 (B) from *M. galloprovincialis* with similar proteins from the most important taxonomic groups. A neighbor-joining (NJ) phylogenetic tree was constructed. The numbers on the branches represent bootstrap values.
(TIF)

Figure S2 NJ trees showing the phylogenetic relationship of the different members of the Bcl-2 family (A) and the six groups of TMBIM proteins (B) from major lineages of organisms, including the representative sequences from invertebrates and vertebrates. The numbers on the branches represent bootstrap values.
(TIF)

Figure S3 NJ phylogenetic tree constructed with Dff-A, Dff-B and CIDE sequences from invertebrates and vertebrates. The numbers on the branches represent bootstrap values.
(TIF)
