# Supplementary figures and images for: Genes of the Mitochondrial Apoptotic Pathway in Mytilus galloprovincialis
Source: PLoS One. 2013 Apr 23;8(4):e61502. doi: 10.1371/journal.pone.0061502 (PMC3634015; doi:10.1371/journal.pone.0061502)

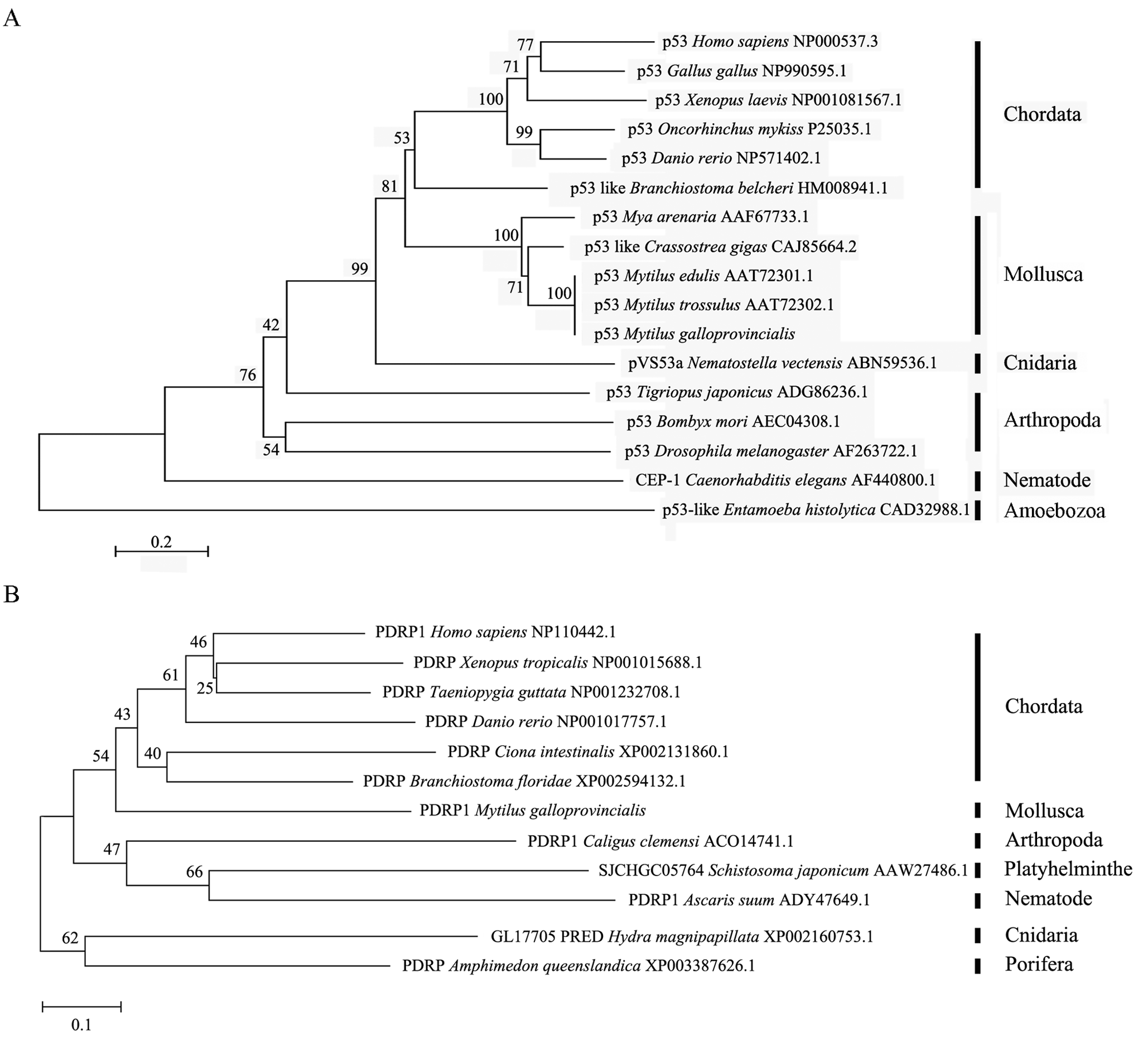

Supplement: Figure S1 — Phylogenetic relationship of p53 (A) and PDRP1 (B) from M. galloprovincialis with similar proteins from the most important taxonomic groups. A neighbor-joining (NJ) phylogenetic tree was constructed. The numbers on the branches represent bootstrap values. (TIF) [file pone.0061502.s001.tif]

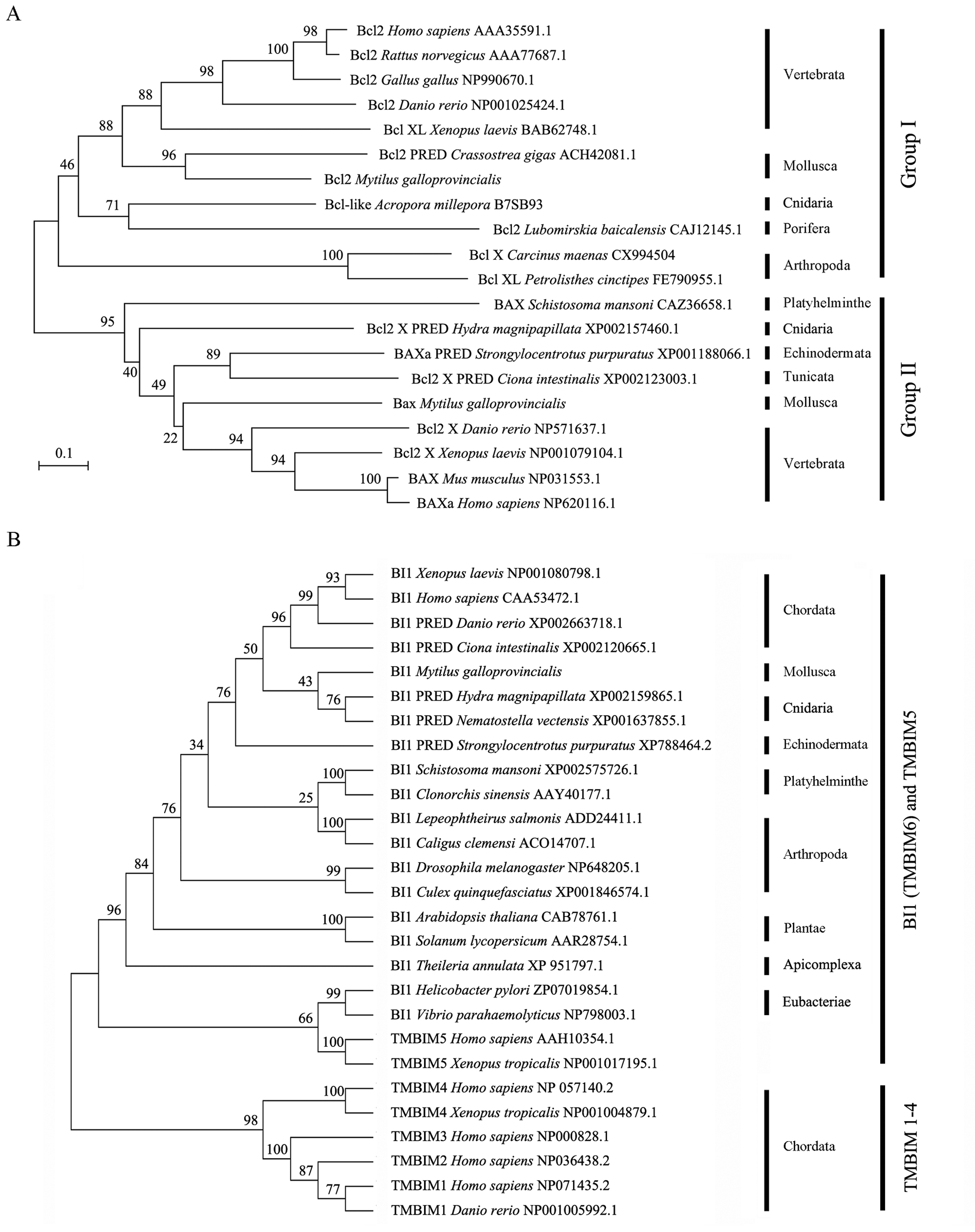

Supplement: Figure S2 — NJ trees showing the phylogenetic relationship of the different members of the Bcl-2 family (A) and the six groups of TMBIM proteins (B) from major lineages of organisms, including the representative sequences from invertebrates and vertebrates. The numbers on the branches represent bootstrap values. (TIF) [file pone.0061502.s002.tif]

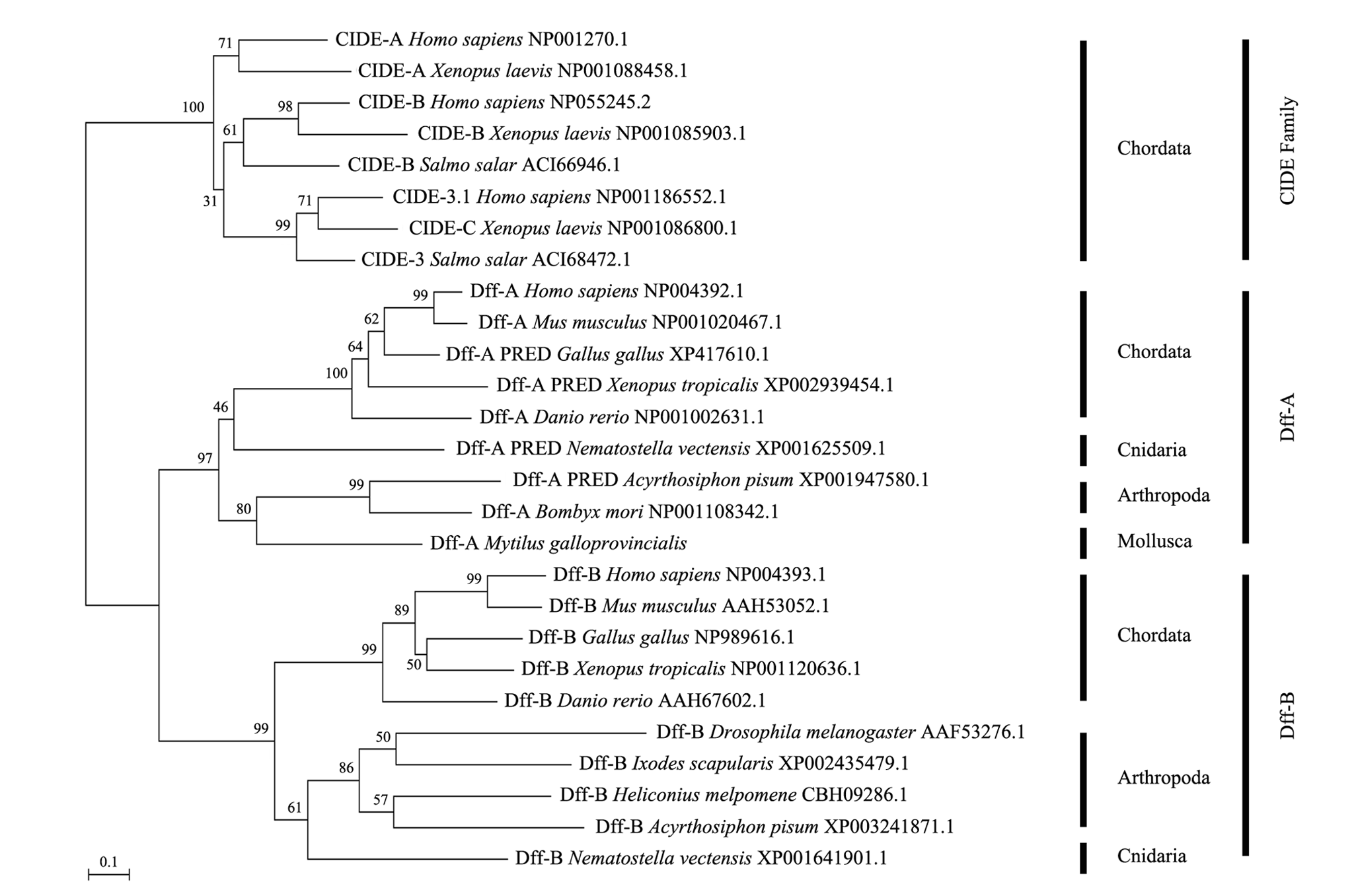

Supplement: Figure S3 — NJ phylogenetic tree constructed with Dff-A, Dff-B and CIDE sequences from invertebrates and vertebrates. The numbers on the branches represent bootstrap values. (TIF) [file pone.0061502.s003.tif]
